# Case Report: Long-term follow-up of a schoolboy with Gitelman syndrome and epilepsy—causation or coincidence?

**DOI:** 10.3389/fped.2026.1772948

**Published:** 2026-03-05

**Authors:** Jiao Xue, Ying Zhang, Hongshan Zhao, Fei Li, Chengqing Yang, Zhi Yi, Kaixuan Liu, Zhenfeng Song

**Affiliations:** 1 Department of Pediatric Neurology, The Affiliated Hospital of Qingdao University, Qingdao, Shandong, China; 2 Department of Anesthesiology, The Affiliated Hospital of Qingdao University, Qingdao, Shandong, China

**Keywords:** epilepsy, Gitelman syndrome, hypokalemia, hypomagnesemia, *SLC12A3* gene

## Abstract

**Introduction:**

Gitelman syndrome (GS) presents with a broad range of clinical manifestations. Although uncommon, seizures secondary to severe metabolic alkalosis or hypomagnesemia have been documented. A concurrent diagnosis of epilepsy in patients with GS is even rarer.

**Case presentation:**

We report the case of a 12-year-old boy whose chief complaint was recurrent convulsions. Initial laboratory evaluation revealed normal serum magnesium levels, which subsequently decreased during follow-up. Persistent hypokalemia, hyperaldosteronism, and hypomagnesemia in subsequent disease course, as well as mutations of the *SLC12A3* gene, confirmed the diagnosis of GS. Based on long-term monitoring of seizure episodes, electroencephalogram findings, and the electrolyte levels during an epileptic seizure, a diagnosis of epilepsy was established. His seizures were well controlled with levetiracetam.

**Conclusion:**

We report a case of GS presenting with convulsions as the chief complaint. The etiology of epilepsy in this case remains unclear and may represent either a causal association or a coincidental comorbidity with GS. The mechanism of the atypical dynamics of serum magnesium levels in this patient—normal levels initially followed by a subsequent decrease—warrants further investigation.

## 1 Introduction

Gitelman syndrome (GS, MIM 263800) is a rare salt-losing tubulopathy characterized by hypokalemic metabolic alkalosis, hypomagnesemia, and hypocalciuria (1). This autosomal recessive disorder results from inactivating mutations in the *SLC12A3* gene, which encodes the thiazide-sensitive sodium–chloride cotransporter located in the luminal membrane of the distal convoluted tubule of the kidney (2). The clinical presentation of GS varies widely, ranging from asymptomatic cases to mild symptoms such as fatigue, nocturia, muscle weakness, or cramps, and more severe manifestations including tetany, paralysis, rhabdomyolysis, or lethal arrhythmia (3). Though rare, convulsions secondary to severe metabolic alkalosis or hypomagnesemia have been documented (4). Even fewer cases have been associated with a diagnosis of epilepsy (5, 6). Here, we report the case of a boy who initially presented with convulsions unrelated to hypokalemia. The diagnosis of GS was confirmed based on persistent hypokalemia and hyperaldosteronism, the development of hypomagnesemia during follow-up, and the identification of *SLC12A3* gene mutations. During long-term follow-up, epilepsy was diagnosed and effectively managed with levetiracetam.

## 2 Case presentation

Our patient was a 12-year-old boy admitted to our hospital for an afebrile convulsion, which was characterized as eyes deviated to right, twitching and slanting of the mouth to the right, lip cyanosis, clenched fists, limbs stiffness, and loss of consciousness lasting several minutes. He was the first child of his non-consanguineous, healthy parents, with normal intelligence and no remarkable family history. On admission, he appeared alert and oriented. The patient’s measurements and vital signs were as follows: body height 142 cm (3th–10th centile), body weight 32 kg (3th–10th centile), temperature 36.7°C, blood pressure 96/65 mmHg, heart rate 92/min, respiratory rate 18/min, and SpO_2_ 100% at room air. Neurological examination—including cranial nerve examination, meningeal irritation signs, and pathologic reflexes—was unremarkable. Serum electrolytes showed serum potassium (K) of 3.0 mmol/L (3.5–5.3 mmol/L), serum sodium (Na) of 137.0 mmol/L (137.0–147.0 mmol/L), serum chlorine (CL) of 94.0 mmol/L (99.0–110.0 mmol/L), serum magnesium (Mg) of 0.77 mmol/L (0.75–1.02 mmol/L), serum calcium of 2.30 mmol/L (2.41–2.52 mmol/L), and serum phosphorus (P) of 1.44 mmol/L (0.85–1.51 mmol/L). Blood ammonia, serum glucose, and blood lactic acid levels were normal. Renal function test showed serum urea of 5.33 mmol/L (2.60–7.50 mmol/L), creatinine (Cr) of 40.00 µmol/L (31.00–132.00 µmol/L), and a blood urea nitrogen/creatinine ratio of 33. Blood gas analysis showed PH 7.45 (7.35–7.45), pO_2_ 101.00 mmHg (83–108 mmHg), pCO_2_ 44.00 mmHg (35–48 mmHg), HCO_3_ 29.30 mmol/L (21–28 mmol/L), and BE 5.70 mmol/L (−2–3 mmol/L). The 24-h urine electrolyte test showed urine potassium 32.67 mmol/24 h (25–100 mmol/24 h), sodium 91.00 mmol/24 h (130–260 mmol/24 h), chlorine 76.00 mmol/24 h (170–250 mmol/24 h), and calcium 0.21 mmol/24 h (2.5–7.5 mmol/24 h). Hypertension-related tests showed activation of the renin–angiotensin–aldosterone system (RAAS) (Table 1). The electrocardiogram (ECG) was normal. Brain magnetic resonance imaging showed no abnormality. Renal and adrenal computed tomography scans showed decreased volume of the left kidney and increased volume of the right kidney. The electroencephalogram (EEG) revealed generalized spike-and-wave discharges, predominantly in bilateral anterior regions.

**Table 1:** Activation of the renin–angiotensin–aldosterone system (RAAS).

Test item	Supine position			Standing position		
Unit	Renin (ng/mL/h)	Angiotensin II (pg/mL)	Aldosterone (pg/mL)	Renin (ng/mL/h)	Angiotensin II (pg/mL)	Aldosterone (pg/mL)
Results	>19.13	109.41	346.57	4.17	88.89	350.7
Reference	0.10–6.56	50.00–120.00	70.00–300.00	0.15–2.33	25.00–60.00	30–160.00

Review of the patient’s medical history revealed two episodes of syncope (specific manifestations not available) at approximately 4 and 5 years of age. During the second syncope episode, he was evaluated at a local clinic and found to have a serum potassium level of 2.9 mmol/L, which improved after symptomatic treatment.

Mutation analysis showed compound heterozygous variants of *SLC12A3* (NM_000339.2): c.961C>T (p.R321W) in exon 7 inherited from his father and c.1288T>G (p.C430G) in exon 10 inherited from her mother. The missense variant c.961C>T was documented as “likely pathogenic” related to GS in ClinVar (https://www.ncbi.nlm.nih.gov/clinvar/variation/1067766/). The missense variant c.1288T>G is conserved evolution. It was predicted by PolyPhen-2 to be “probably damaging” on protein function with a probabilistic score of 1.000 and by MutationTaster to be “disease causing” with a probabilistic score of 0.999.

Based on his clinical presentation, physical examination, auxiliary examination, and gene analysis results, the patient was diagnosed with GS and initiated on oral potassium chloride sustained-release tablet. After discharge, he continued to take potassium chloride orally. Fluctuations were noted in the following levels during dynamic review: serum potassium fluctuated 3.5–3.9 mmol/L, serum sodium 137–142 mmol/L, serum chlorine 92–96 mmol/L, and serum magnesium 0.63–0.66 mmol/L. At the 2- and 3-month follow-up, the patient again had convulsions and sought treatment at the local emergency department. His serum electrolytes showed serum potassium of 3.6 and 3.5 mmol/L (3.5–5.3 mmol/L), serum sodium of 138 and 140 mmol/L (137.0–147.0 mmol/L), serum chlorine of 93 and 96 mmol/L (99.0–110.0 mmol/L), and serum magnesium of 0.64 and 0.66 mmol/L (0.75–1.02 mmol/L), respectively. His ECG was normal. Combined with his EEG results, he was considered to be suffering from epilepsy, and levetiracetam was added to his treatment regimen. He did not suffer from convulsions during the following 3 years, after which levetiracetam was gradually withdrawn. At present—3 years hence—he has been seizure-free without the aid of any antiseizure medication. However, he continues to take oral potassium chloride tablets, which help maintain his electrolyte levels within the normal range. His height and weight are in the 50th percentile for his age group.

## 3 Discussion

GS typically presents with non-specific symptoms such as weakness, fatigability, and polyuria. Seizures are a rare manifestation and are classically associated with hypokalemia, hypomagnesemia, hypocalciuria, hyperreninemia, and hyperaldosteronism (7). However, GS exhibits considerable genetic heterogeneity and phenotypic diversity, which varies not only among individuals with different genetic variants but also among family members carrying the same mutation (8). Patients with GS may be asymptomatic, with hypokalemia occasionally being found during routine physical examination; however, GS patients may also present with weakness, fatigability, and seizures as the main manifestations (7–9).

GS is diagnosed based on clinical symptoms, biochemical abnormalities, and genetic testing (8). Hypokalemia and hypomagnesemia represent the most characteristic metabolic alterations in GS. In the present case, the diagnosis of GS was confirmed by the presence of hypokalemia and hyperaldosteronism, along with the detection of *SLC12A3* gene variants. Notably, however, the patient exhibited a normal serum magnesium level at initial presentation, which subsequently declined during the course of the disease. Normal magnesium levels in GS have also been reported in previous studies. For instance, Zhang et al. (8) observed that 31 Chinese pediatric patients with GS presented with hypokalemic metabolic alkalosis and hyperaldosteronism, while 5/31 (16.1%) patients displayed normal or even elevated serum magnesium levels. These findings suggest that hypomagnesemia may not be an obligatory criterion for diagnosis. Therefore, in clinically suspected cases without hypomagnesemia, genetic testing should be performed to establish a definitive diagnosis.

Uncommonly, our patient presented with seizures as the main complaint. As a rare presentation of GS, one possible pathophysiological mechanism of seizure might be hypomagnesemia (10). Avoli et al. (11) and Mody et al. (12) suggested that hypomagnesemia may diminish the inhibitory effect of magnesium on glutamate-mediated depolarization, thereby increasing neuronal excitability and seizure susceptibility. In our patient, however, serum magnesium levels were within the normal range at the time of the initial seizure. This observation is supported by a report from Shahzad et al. (7), which described a patient who experienced generalized tonic–clonic seizures despite normal serum magnesium levels. The pathological mechanism underlying seizures in GS patients without hypomagnesemia remains unclear. It is noteworthy that our patient experienced several subsequent seizures. Given the presence of abnormal EEG findings, the possibility of incidental epilepsy—rather than as a direct consequence of GS—cannot be excluded. Our patient’s favorable response to levetiracetam further supports the diagnosis of epilepsy. Although rare, epilepsy has been reported as an extrarenal complication of GS (6). Fujimura et al. (6) reported that three out of 121 (2.5%) GS patients were diagnosed with epilepsy, suggesting that GS may increase susceptibility to seizure disorders.

In conclusion, we have reported a case of GS in a boy presenting with convulsions as the chief complaint. The initial serum magnesium level was within the normal range, slightly decreasing thereafter during the course of the disease. Subsequent investigations and long-term follow-up confirmed the diagnosis of both GS and epilepsy. The etiology of epilepsy in this case remains unexplained and may represent either a causal association or a coincidental comorbidity with GS. Furthermore, the mechanism of the atypical dynamics of serum magnesium levels in this patient—normal levels initially followed by a subsequent decrease—warrants further investigation.

## Data Availability

The original contributions presented in the study are included in the article/Supplementary Material, further inquiries can be directed to the corresponding author.
